# SARS-CoV-2–Legionella Co-Infections: A Systematic Review and Meta-Analysis (2020–2021)

**DOI:** 10.3390/microorganisms10030499

**Published:** 2022-02-23

**Authors:** Matteo Riccò, Pietro Ferraro, Simona Peruzzi, Alessandro Zaniboni, Silvia Ranzieri

**Affiliations:** 1 AUSL–IRCCS di Reggio Emilia, Servizio di Prevenzione e Sicurezza Negli Ambienti di Lavoro (SPSAL), Local Health Unit of Reggio Emilia, 42122 Reggio Emilia, Italy; 2 Servizio di Medicina del Lavoro, ASL di Foggia, 71121 Foggia, Italy; dott.pietro.ferraro@gmail.com; 3 AUSL–IRCCS di Reggio Emilia, Laboratorio Analisi Chimico Cliniche e Microbiologiche, Ospedale Civile di Guastalla, 42016 Guastalla, Italy; simona.peruzzi@ausl.re.it; 4 Department of Medicine and Surgery, University of Parma, 43126 Parma, Italy; alessandro.zaniboni@unipr.it (A.Z.); silvia.ranzieri@unipr.it (S.R.)

**Keywords:** *Legionella pneumophila* typing, Legionnaires’ disease, SARS-CoV-2, COVID-19, diagnosis, epidemiology

## Abstract

Legionnaires’ Disease (LD) is a severe, sometimes fatal interstitial pneumonia due to *Legionella pneumophila*. Since the inception of the SARS-CoV-2 pandemic, some contradictory reports about the effects of lockdown measures on its epidemiology have been published, but no summary evidence has been collected to date. Therefore, we searched two different databases (PubMed and EMBASE) focusing on studies that reported the occurrence of LD among SARS-CoV-2 cases. Data were extracted using a standardized assessment form, and the results of such analyses were systematically reported, summarized, and compared. We identified a total of 38 articles, including 27 observational studies (either prospective or retrospective ones), 10 case reports, and 1 case series. Overall, data on 10,936 SARS-CoV-2 cases were included in the analyses. Of them, 5035 (46.0%) were tested for *Legionella* either through urinary antigen test or PCR, with 18 positive cases (0.4%). A pooled prevalence of 0.288% (95% Confidence Interval (95% CI) 0.129–0.641), was eventually calculated. Moreover, detailed data on 19 co-infections LD + SARS-CoV-2 were obtained (males: 84.2%; mean age: 61.9 years, range 35 to 83; 78.9% with 1 or more underlying comorbidities), including 16 (84.2%) admissions to the ICU, with a Case Fatality Ratio of 26.3%. In summary, our analyses suggest that the occurrence of SARS-CoV-2–*Legionella* infections may represent a relatively rare but not irrelevant event, and incident cases are characterized by a dismal prognosis.

## 1. Introduction

During 2020, non-pharmacological interventions (NPI) were instrumental in managing the earlier stages of the coronavirus disease (COVID-19) pandemic caused by the severe acute respiratory syndrome coronavirus 2 (SARS-CoV-2) [1,2,3,4]. NPI have been defined as public health measures actions, apart from getting vaccinated and taking medicine, that people and communities can take to help slow the spread of illnesses that aim to prevent and/or control the pathogen’s transmission in the community [5,6]. Available reports suggest that NPI have impacted not only the transmission of the targeted SARS-CoV-2 but also other pathogens. For example, Ullrich and colleagues identified a stark reduction for incident cases of respiratory diseases (from −86% for measles to −12% for tuberculosis), gastrointestinal diseases (from −83% for rotavirus gastroenteritis to −7% for yersiniosis), and even imported vector-borne diseases (from −75% dengue fever to −73% malaria) [2]. On the contrary, early national reports for Legionnaires’ Disease (LD) suggest a more limited effect on the epidemiology of respiratory syndromes caused by Gram-negative bacilli from the genus *Legionella* [7,8,9,10,11,12,13].

LD is a severe, sometimes fatal interstitial pneumonia, with characteristic common extrapulmonary manifestations (i.e., renal failure, encephalopathy, pericarditis), that shares several clinical features with lung infections caused by pneumococcus and other bacteria [8,9]. Before the inception of the SARS-CoV-2 pandemic, the notification rates for LD had sharply increased both in North America [13] and the European Union/European Economic Area (i.e., from 1.2 to 1.8 cases per 100,000 inhabitants between 2011 and 2018) [14,15,16]. As LD has no interhuman spreading, physical distancing was hardly able to contain the spread of this pathogen at the community and/or hospital level. In fact, the small but consistent reduction in notification rates that has been reported by the European Centre for Disease Prevention and Control (ECDC) for European Union Countries since the inception of the SARS-CoV-2 pandemic (i.e., 2.0 per 100,000 person compared to 2.2 per 100,000 person in 2019) substantially reflects the reduction in LD travel-associated cases following the travel ban, and subsequent limitations globally implemented for international travels during the early stages of the pandemic, which were progressively lifted [10,11,12,13].

Even though the majority of SARS-CoV-2 cases only develop mild symptoms [17,18,19], a substantial share of them evolve into acute respiratory failure and require intensive care, including intensive care units (ICU) and mechanical ventilation, with a dismal prognosis. According to available data, 90% of the critically ill patients with severe SARS-CoV-2 pneumonia receive empiric antibiotic treatment upon ICU admission [20,21,22], and early reports have suggested high rates of bacterial co-infections [23,24,25,26], including the *Legionella* species [27], with a poor prognosis [28,29]. For example, in an early study from Xing et al. [27], the prevalence rate for SARS-CoV-2-*Legionella* co-infections peaked at an unprecedented 20.0% in 30 ICU patients. Even though this specific report was affected by several potential shortcomings (e.g., the reduced number of participants and the detection of *Legionella* through IgM-specific antibodies), risk factors for complicated LD and severe COVID-19 overlap (i.e., age > 65 years, male sex, smoking history, chronic lung disease, diabetes, and various conditions associated with immunodeficiency, including solid or blood cancer, transplantation, and/or chemotherapy) [8,9,30,31,32,33]. Moreover, the early stages of these disorders are quite similar, with fever, headache, confusion, dyspnea, nausea, and gastrointestinal symptoms [34]. In other words, co-infections SARS-CoV-2–*Legionella* may represent a significant clinical issue because of their alleged frequency; the difficult differential diagnosis, particularly at the community level [35,36]; and the potentially dismal prognosis.

Our aim was therefore to perform systematic review and meta-analysis in order to summarize the available data about the risk of co-infections SARS-CoV-2–*Legionella*, specifically focusing on their prevalence, risk factors, and two main outcomes—ICU admission and case fatality ratio (CFR).

## 2. Materials and Methods

We performed a systematic review according to the PRISMA (Prepared Items for Systematic Reviews and Meta-Analysis) guidelines [37,38]. More precisely, we searched two scientific databases (i.e., PubMed and EMBASE) for relevant studies until 25 December 2021, without any chronological restriction. Despite their potential significance, preprints were ultimately excluded from the analyses when they were not peer-reviewed. A search strategy was defined through a combination of the following keywords (free text and Medical Subject Heading [MeSH] terms): (“Legionnaire disease” or “Legionella” or “Legionellosis” or “Legion*”) and (“SARS-CoV-2” or “COVID” or “coronavirus”), including only documents written in any of the languages spoken by the investigators (i.e., Italian, English, German, French, Spanish).

Documents potentially eligible for review after the initial inquiry were both prospective and retrospective observational studies, including case studies and case reports on the occurrence of new diagnoses of LD in individuals affected by SARS-CoV-2 infections, irrespective of the clinical settings (i.e., community, hospitals, or nursing homes). Two authors (S.P. and P.F.) initially screened the titles of retrieved articles for relevance to the subject. Documents that met this initial requirement were then excluded if: (1) full text was not available, (2) articles were written in a language not understood by the reviewers, (3) reports lacked information about the timeframe, (4) diagnostic procedures performed for *Legionella* spp. were not clearly reported, (5) reports lacked definition of the geographical settings, or it was only vaguely defined. All other documents were retained for full-text review and subsequent analyses. Full-text versions of eligible articles were independently assessed by two investigators. Disagreements were then resolved by consensus between the two reviewers. Where they did not reach consensus, input from the chief investigator (MR) was obtained. Further studies were retrieved from reference lists of relevant articles and consultation with experts in the field.

The studies can be summarized as follows:

(a) Case-control studies, cross-sectional studies, and cohort studies

Data abstracted included: (a) settings of the study: prevalence year, country, design of the study (prospective vs. retrospective); (b) settings of the report (i.e., intensive care unit (ICU), non-ICU, or both); (c) total number of SARS-CoV-2 positive cases (i.e., reference population); (d) total number of individuals sampled for LD; (e) diagnostic items for *Legionella* spp. (i.e., *Legionella* urinary antigen test (LUAT) vs. other procedures); (f) number of cases with a positive specimen for *Legionella* spp.; (g) main demographics of sampled cases (age and gender, where available).

First, a descriptive analysis was performed to report the characteristics of the included studies, with a calculation of the crude figures. Pooled prevalence estimates were then calculated by means of prevalent cases per 100 SARS-CoV-2 cases. To cope with the presumptive heterogeneity of the sampled studies, we opted for the random effects model. The amount of inconsistency between the included studies was estimated by means of the I^2^ statistic (i.e., the percentage of total variation across studies that is due to heterogeneity rather than chance). According to current understanding, I^2^ values were categorized as follows: 0–25%, low heterogeneity; 26–50%, moderate heterogeneity; ≥50%, substantial heterogeneity. To investigate publication bias, contour-enhanced funnel plots representing the Egger test for quantitative publication bias analysis (at a 5% of significance level) were generated. Radial plots were then calculated and visually inspected to rule out small study bias. The relationship between the number of LD cases, and the individual (i.e., share of male gender, median age) and sampling data (i.e., timeframe, geographic area, diagnostic procedure for *Legionella* infection, clinical settings, sampling rate over incident cases) were included in a Poisson regression model, with calculation of correspondent Incident Rate Ratios (IRR) and 95% Confidence Intervals (95% CI).

(b) Case reports and case series

First, individual characteristics and settings of the case(s) were retrieved, including the year and country where the case(s) occurred; age and gender; and any of the following underlying conditions: obesity, hypertension, steroid therapy, smoking history, cardiovascular disease, asthma, chronic respiratory disease (other than asthma), chronic kidney disease, diabetes, or cancer (solid or blood cells). Through the analysis of the text, the following information regarding the infection(s) were also collected: presumed or confirmed source(s) of infections for both SARS-CoV-2 and *Legionella pneumophila* (e.g., community, hospital, travel, etc.); eventual admission to ICU, with and without intubation, and their respective length; outcome (i.e., recovery, ongoing disease, death).

The collected data were then summarized, and the association of categorical variables, with the two outcomes represented by admission to the ICU (yes vs. no) and death (yes vs. no), was initially calculated using Fisher’s exact test.

All calculation were performed on R 4.0.3 (R Core Team (2020). R: A language and environment for statistical computing. R Foundation for Statistical Computing, Vienna, Austria, https://www.R-project.org/ [39] using packages epiR (v. 2.0.19), EpiReport (v 1.0.1), fmsb (0.7.0), msm (1.6.8), sandwich (3.0-0), meta (4.9-9).

*Ethical approval*. No ethical approval was needed for this study, as no individual data were identifiable, and only aggregated data were analyzed and presented.

## 3. Results

**Figure 1:**
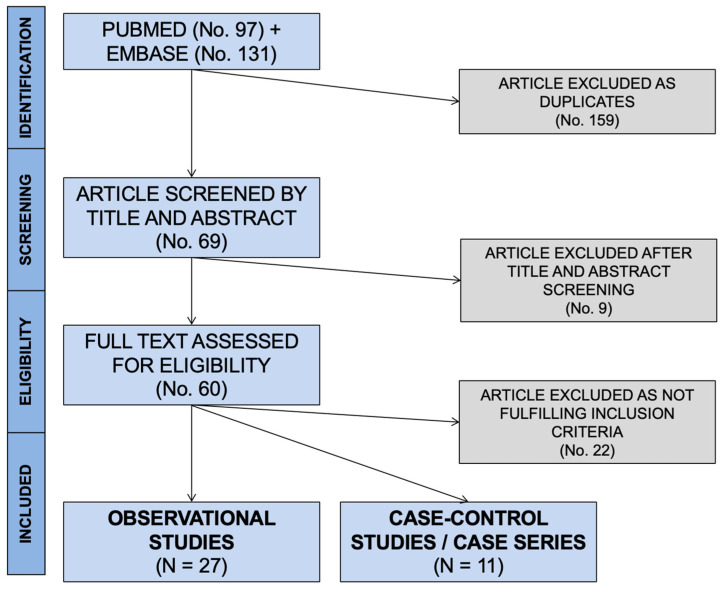
PRISMA flowchart for retrieved studies.

Initially, 228 entries were identified, including a total of 97 abstracts from PubMed and 131 from EMBASE. As 159 were duplicated across the sources, 69 entries were initially screened (Figure 1).

After applying the inclusion and exclusion criteria (Figure 1), a total of 38 articles were included in the qualitative synthesis [23,24,26,28,29,35,40,41,42,43,44,45,46,47,48,49,50,51,52,53,54,55,56,57,58,59,60,61,62,63,64,65,66,67,68,69,70,71,72]. More precisely, 27 observational studies (see Table 1 for details), 1 case series, and 10 case reports (see Appendix A for details) were included. As one of the cross-sectional studies [62] included detailed data on the only case reported, the corresponding data were summarized alongside the case reports.

### 3.1. Observational Studies

The 27 retrieved studies included a total of 10,936 cases positive for SARS-CoV-2, all hospitalized because of COVID-19. From these index cases, 5035 specimens were collected to assess the co-infection by *Legionella pneumophila* (46.0%; Table 1). The sample size ranged from a minimum of 20 [63] to a maximum of 1396 patients [56].

The corresponding share of sampled patients ranged from 2.1% [59] to 100% [27,42,43,45,48,51,53,58,62,63] of index cases. In most cases, their status was assessed with Urinary *Legionella* Antigen Testing (ULAT) (76.7%), but a substantial share of them was also assessed with PCR on bronchial specimens (23.3%). The majority of the cases were males (61.6%, range: 42.6–79.0%) [65,73], and median age was 61 years.

**Figure A1:**
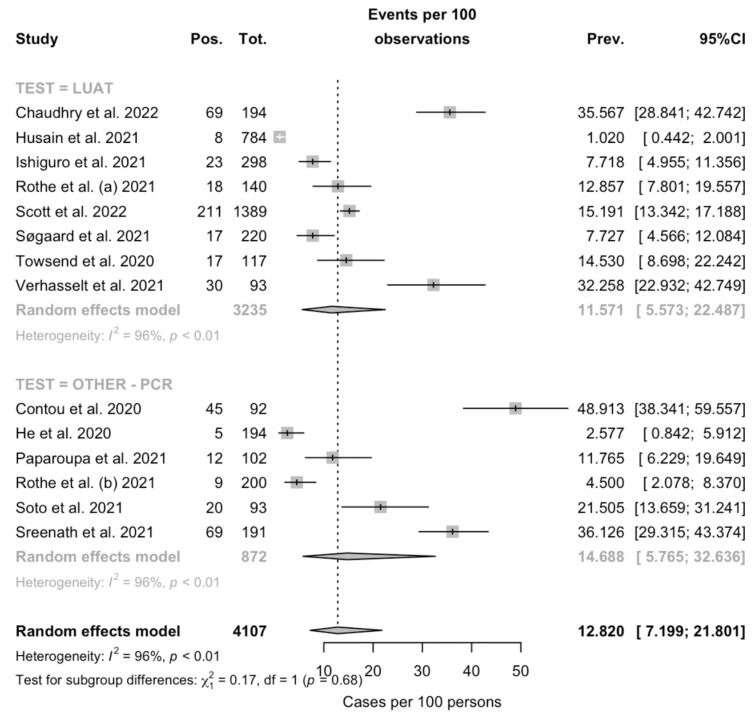
Forest plot representing the estimated case fatality ratio (CFR) among individuals affected by SARS-CoV-2 in the retrieved studies. Pooled CFR was estimated in 12.820% (95% Confidence Interval (95% CI) 7.199–21.801), with estimates that were considerably greater in PCR-based studies (14.688%, 95% CI 5.765–32.636) compared to those based on LUAT (11.571%, 95% CI 5.573–22.487) (note: Pos. = deaths; Tot. = total cases; Prev. = Estimated CFR).

**Figure A2:**
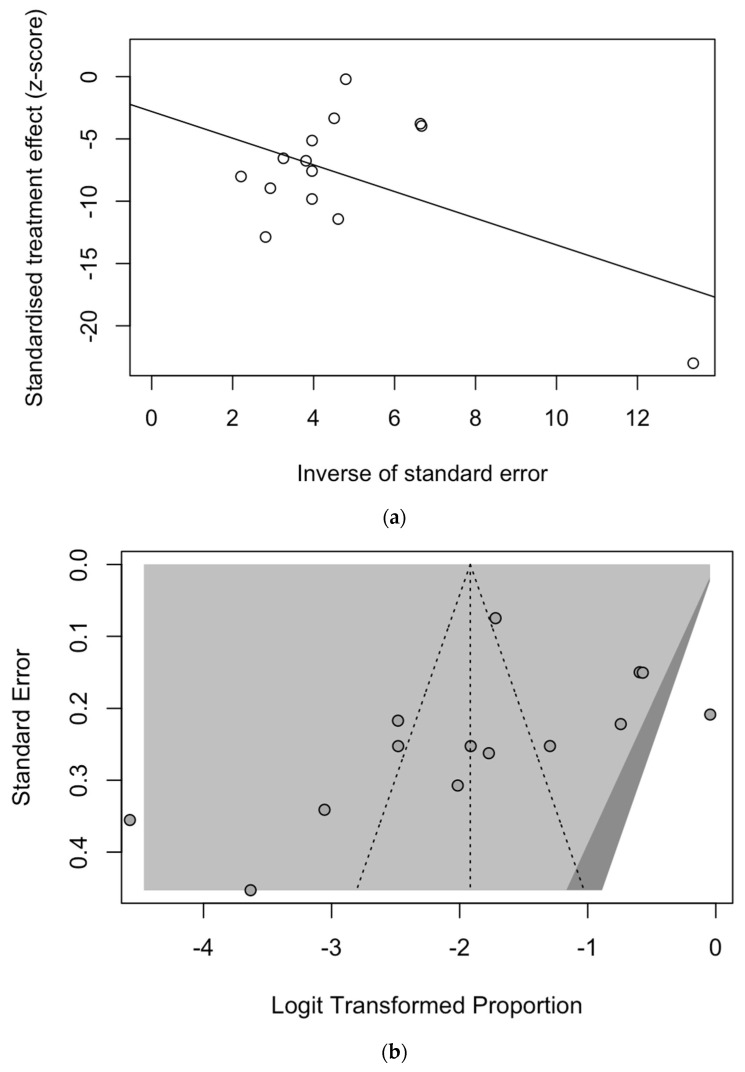
Border-enhanced funnel plot for studies included in the meta-analysis (**a**), and the corresponding radial plot (**b**). Visual inspection of the contour-enhanced funnel plot suggested substantial evidence of publication bias, but this was substantially ruled out by the Egger test (i.e., t = −1.05, df = 12, *p*-value = 0.3131). On the other hand, in radial plots, the studies were substantially scattered across the regression line, suggesting no significant small study effect.

In the majority of the reported studies, index cases were recruited from both normal wards and ICUs, while 6 studies (22.2%) only included patients from ICU [23,24,41,51,53,63]. Data on the actual number of admissions to ICU were available from 20 out the 27 studies, with a cumulative share of 47.4% (range: 6.6–100%). Mortality rates of patients sampled for *Legionella* were available in only 14 studies, allowing a pooled estimate case fatality ratio of 12.5% (95% CI 6.9–21.4) (See Appendix A Figure A1 and Figure A2).

As shown in Table 2, the majority of the observational studies were of retrospective design (92.6%) and reported on cases occurring during the “first wave” of the pandemic (21 studies, 77.8%), including a total of 9073 index cases of SARS-CoV-2 (83.0%). The majority of the reports came from European countries (n = 17, 63.0%), followed by China (n = 4, 14.8%) and India (n = 2, 7.4%), while a single report was retrieved from Israel, Japan, Peru, and the USA, respectively (3.7% each).

Overall, 18 cases of co-infections were retrieved (0.4% of total samples), with the majority of studies (18 out of 27, 66.7%) not reporting a single occurrence [23,24,26,42,44,46,48,50,53,54,55,56,58,59,61,63]. The majority of diagnoses was obtained from studies performed during the “first wave” (61.1%) and in European countries (50.0%).

However, assuming the occurrence of *Legionella* infections in studies from the “first wave” as a reference category, an increased Risk Ratio (RR) was identified for the studies performed in the subsequent months (RR 6.508, 95% CI 1.909–22.190). On the contrary, considering European studies as a reference, no differences were identified for other geographic areas. Similarly, neither the study design (prospective vs. retrospective RR 1.864, 95% CI 0.250–13.920) nor diagnostic procedures (PCR vs. LUAT RR 1.324, 95% CI 0.497–3.521) exhibited any substantial difference in new diagnoses of SARS-CoV-2 and *Legionella* co-infection.

**Figure 2:**
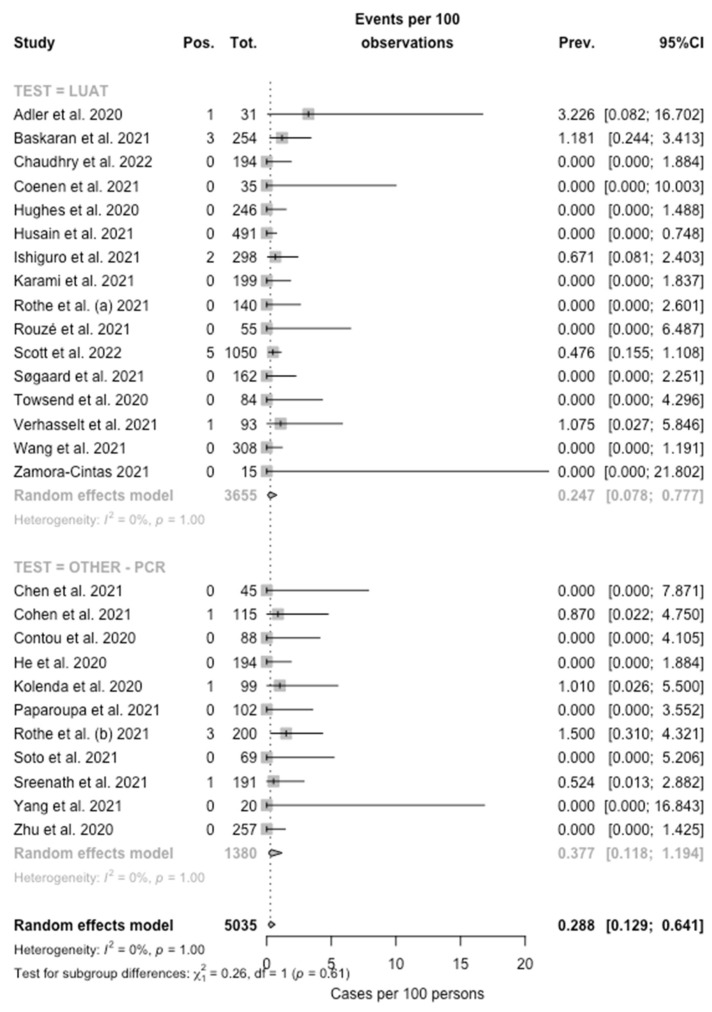
Forest plot representing the estimated pooled prevalence (Prev.) for *Legionella* infection (Pos.) among individuals affected by SARS-CoV-2 (Tot.). The pooled prevalence rate was estimated at 0.288% (95% Confidence Interval (95% CI) 0.129–0.641), with estimates that were considerably greater in PCR-based studies (0.377%, 95% CI 0.118–1.194) compared to those based on LUAT (0.247%, 95% CI 0.078–0.777).

When the collected data were pooled, the eventual prevalence for *Legionella* was then estimated in 0.288% (95% CI 0.129–0.641), with low heterogeneity (I^2^ = 0%, Q = 32.50, τ^2^ = 0.446, *p* = 1.00) (Figure 2) being substantially greater among studies based on PCR testing (0.377%, 95%CI 0.118–1.194) compared to those based on LUAT (0.247%, 95% CI 0.078–0.777; chi squared = 7029.8, *p* < 0.001).

**Figure 3:**
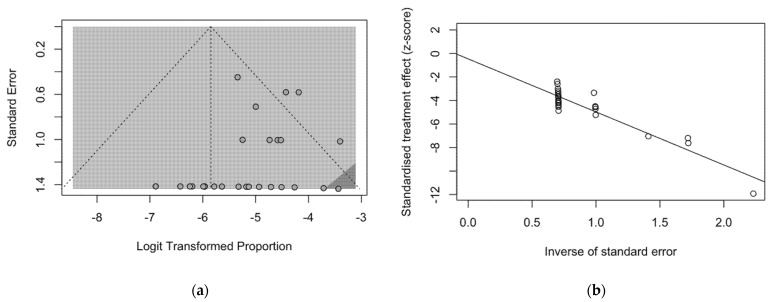
Border-enhanced funnel plot for studies included in the meta-analysis (**a**), and the corresponding radial plot (**b**). Visual inspection of the contour-enhanced funnel plot suggested substantial evidence of publication bias, and this was substantially excluded by the Egger test (i.e., t = −1.25, df = 25, *p*-value = 0.2235). In fact, in radial plots, the studies on were substantially scattered across the regression line, suggesting no significant small study effect.

The presence of publication bias was evaluated using funnel plots and the regression test for funnel plot asymmetry, as shown in Figure 3. First, the effect sizes of the studies were plotted against their standard errors, and the visual evaluation of the funnel plot suggested a possible publication bias (i.e., publication or non-publication of relevant trials, depending on the nature and direction of the results) (Figure 3a). This subjective evidence was substantially ruled out after the regression test (Egger test: t = −1.25, df = 25, *p*-value = 0.2235). Moreover, in the radial plot (Figure 3b), individual estimates were substantially scattered across the regression line, substantially ruling out that smaller studies showed different outcomes than large ones, which may threaten the validity of estimates (i.e., small study effect).

Eventually, data on the reported cases of SARS-CoV-2–*Legionella* co-infections were included as outcome variables in a Poisson regression model having the timeframe of the study (i.e., “first wave” vs. subsequent studies), geographical (Europe vs. other areas), and clinical settings (i.e., normal wards and ICU vs. ICU-only studies), diagnostic procedure (i.e., PCR vs. LUAT), median age of the study participants, share of male individuals, and sampling rates as covariates. As shown in Table 3, a timeframe corresponding to the “first wave” of SARS-CoV-2 pandemic (IRR 0.796, 95% CI 0.735–0.863), geographic setting other than Europe (IRR 0.609, 95% CI 0.564 to 0.658), and the use of LUAT instead of PCR testing (IRR 0.688, 95% CI 0.622 to 0.760) were characterized as negative effectors for new diagnoses of SARS-CoV-2–*Legionella* co-infections. On the contrary, a design study only including patients from ICUs (IRR 9.009, 95% CI 6.923 to 11.725) and increased sampling over incident SARS-CoV-2 cases (+1%, IRCC 1.042, 95% CI 1.039 to 1.044) was identified as strong positive effectors. Similarly, patients’ characteristics, such as age (+1 year, IRR 1.055, 95% CI 1.047 to 1.062) and male gender (+1% in the sampled population, IRR 1.119, 95% CI 1.112 to 1.125), were associated with an increased occurrence of SARS-CoV-2-*Legionella* co-infection.

### 3.2. Case Reports and Case Series

A total of 19 cases were ultimately retrieved: 11 from individual case reports (57.9%), 6 from a single case series included in a larger report based on French national data, and a further case that was individually and accurately described in a retrospective study from Germany. The detailed summary of the retrieved cases is reported in Appendix A Table A1.

Briefly (see Table 4), the majority of cases were males (84.2%), with a mean age of 61.9 years ± 16.1 (range 37–83). Overall, 78.9% of cases had at least one pre-existing risk factor, represented by cardiovascular diseases (31.6%), smoking history and steroid therapy (both 26.3%), diabetes (21.1%), cancer and obesity (both 15.8%). The majority of cases were reported from France (seven cases, 36.8%) [35], followed by Japan and the UK (two cases, 10.5%) [28,68,70], while a single case was reported from Chile, Germany, Italy, Portugal, Saudi Arabia, Spain, and the USA, respectively [29,62,64,65,66,67,69,71]. In the majority of reports, both SARS-CoV-2 and *Legionella* infection seemingly occurred at the community level, i.e., no specific causes were identified (68.4%), followed by travel (15.8%) and hospitals (10.5%), with analogous estimates for both pathogens. In two cases, multiple causes of exposure were identified, including the exposure to plumbing systems [35,70].

The majority of reported cases required intensive care at an ICU (84.2%), with a total stay that ranged between 5 and 27 days (average: 17.7 days ± 8.0). Unfortunately, data on mechanical ventilation at the ICU were not consistently reported across the individual reports. Regarding the eventual outcome, 47.4% were discharged either at home or in non-intensive wards, and 26.3% were still treated in ICU at the time of the report. A total of five deaths were reported (case fatality ratio of 26.3%), with all deaths occurring in individuals aged 65 years or older (Table 5).

## 4. Discussion

In our estimates, *Legionella pneumophila* was identified in 0.288% (95% CI 0.129–0.641) of sampled SARS-CoV-2 patients. Compared to other potential co-infections (e.g., *Candida* spp.; *Pseudomonas* spp.; *Staphilococci*, and Enterococci) that may peak at up to 20% of incident cases, the potential occurrence of *Legionella* infections may therefore appear quite insignificant [42,43,74], and substantially lower than that addressed by early reports from mainland China [27]. Moreover, it should be stressed that nearly half of the sampled cases were from ICUs (i.e., 47.4%), an estimate that exceeds most of the available reports for SARS-CoV-2 infections, even for the “first wave,” [75,76], when Grasselli et al. reported a utilization of ICU equal to 16% of all hospitalizations [76]. In this regard, including only ICU cases was characterized as the single most significant effector for new diagnoses (IRR 9.009, 95% CI 6.923–11.725). In other words, the available studies presumptively oversampled patients with more severe COVID-19. In such a setting, bacterial co-infection and a poor prognosis are probably more common than in the majority of SARS-CoV-2 cases from the general population [13,47,75]. Not coincidentally, the risk factors associated with more severe SARS-CoV-2 cases, such as male gender and older age, were identified as significant effectors for new diagnoses of co-infections.

In other words, our figures may represent a substantial overestimate of the real-world epidemiology of SARS-CoV-2–*Legionella* co-infections. Not coincidentally, in the analysis of individual cases, the majority of co-infections required intensive care, with ICU admission (i.e., 84.2% in the corresponding estimates), intubation, and mechanical ventilation. The case fatality ratio appears quite significant, accounting for 26.3% of cases. In addition, a similar share of patients was still receiving medical care when the case was reported, suggesting that co-infection cases may lead to a more unfavorable outcome, with a longer requirement of intensive care. Among the main risk factors for both *Legionella* and SARS-CoV-2 infections (such as obesity, hypertension, steroid therapy, smoking history, cardiovascular diseases, chronic respiratory diseases, chronic kidney diseases, diabetes, cancer, and asthma) [9,16,73,77], only age ≥ 65 years at the time of diagnosis was associated with the eventual death of the patients (*p* = 0.045), as all of deceased patients belonged to this age group. However, no distinctive risk profile was identified.

However, the aforementioned estimates should be cautiously and critically assessed for several reasons.

First, the reported observational studies were not specifically designed for addressing the specific topic of SARS-CoV-2–*Legionella* co-infections, and only a small fraction was designed to assess the occurrence of atypical pathogens [42]. In most cases, the diagnoses were obtained as a consequence of a larger screening, and particularly among reports based on PCR [23,47,49], presumptively as a part of larger differential diagnosis approach.

Second, although the cumulative prevalence of 0.288% in the entirety of SARS-CoV-2 cases may appear quite irrelevant compared to other pathogens, because of the cumulative figures of the pandemic, it would correspond to an unprecedent number of diagnoses for *Legionella* infections in the general population. For instance, since the inception of the SARS-CoV-2 pandemic, the three most populated EU countries (i.e., Germany, France, and Italy) have reported several million cases of SARS-CoV-2 infection (i.e., by 31 December 2020: 1,719,737 cases for Germany, 2,600,498 for France, and 2,107,166 for Italy) [13,78,79,80]. Assuming a 0.288% prevalence over incident SARS-CoV-2 cases, it leads to an estimated burden of *Legionella* infection for the time period from March to December 2020 that exceeds several times the official notification rates for 2020 (i.e., 3818 cases vs. 1281 for Germany, 5773 cases vs. 1328 for France, 4678 vs. 2074 for Italy) [10,11,12]. On the contrary, available data suggest that during 2020 notification rates for *Legionella* have slightly but substantially decreased in most of European countries, presumptively as a consequence of the travel ban implemented during the “first wave” of the pandemic, and the subsequently enforced restrictions to international travels [28,34,68,81].

As *Legionella* infections are notoriously underestimated [9,16,77,82], a possible explanation for the high occurrence among SARS-CoV-2 patients compared to the general population may be found in the increased referral to diagnostic procedures in early stages of the infection, with innovative and more accurate items. In fact, a 30% sales increase for LUAT was reported for 2020 [11], suggesting that more patients were specifically assessed for *Legionella* infections than before, with a similar increasing number of notified cases. Moreover, in our estimates, a large share of individuals was sampled by means of PCR testing, a procedure that is substantially uncommon in the general population [16], and that was associated with an increased occurrence of the diagnosis of co-infections.

Third, it is unclear how many of the sampled individuals had previously received or had not received a large-spectrum antibiotic therapy able to specifically target *Legionella* [83]. As macrolides have been extensively delivered to SARS-CoV-2 patients during the early stages of the pandemic [22,48,84,85,86], we cannot rule out that a significant share of actual co-infections with signs and symptoms of atypical pneumonia may have benefited from early antimicrobial courses [22,85,87], with their eventual impact on *Legionella* infections.

Even the high lethality we were able to identify may represent a substantial overestimate. In fact, only half (i.e., 14 out 27) of the observational studies reported the overall mortality for the entirety of the patients sampled for *Legionella* [23,42,43,48,49,50,52,53,54,55,57,58,60,62]. Even in these reports, a specific analysis of mortality among co-infections of SARS-CoV-2–*Legionella* is lacking, eventually impairing a summary analysis. Therefore, our estimate of 26.3% was based on individual cases. By their design, such reports usually include cases having unusual or novel occurrences, potentially oversampling patients with more severe complications and higher risk for eventual death [88,89]. In this regard, it is important to stress that fort he estimates from the observational studies reported on cases of documented *Legionella* infections, the case reports deliberately focused on patients having an extensive respiratory involvement with atypical pneumonia. Whereas cases of *Legionella* infections occurring among healthy and young individuals may by lost by official reports because of milder clinical features, complicated cases requiring intensive medical care are more easily identified and reported [10,16]. The progressive reduction in the CFR for LD in most developed countries has been similarly explained, i.e., a consequence of the increased diagnosis of milder cases over the more severe ones [10,16,80]. In other words, the overall estimates may have been inflated by an undefined share of patients that have been temporarily colonized by *Legionella* but will not develop the eventual clinical syndrome. However, those from individual case report reflect cases that have a higher risk for complications and mortality from the beginning. Their dismal prognosis therefore reflects the substantial overlapping of two pathogens that mainly target the same organs and tissues through two different strategies.

*Limits*. Despite the potential interest, our study is affected by several limitations. First, our estimates are highly dependent on the parent studies [90,91], being affected by their quality and residual heterogeneity [91]. In this regard, while the quality of the studies we were able to retrieve was quite erratic, particularly in terms of data reporting and sampling strategy, the heterogeneity was scarce. A potential but also systematic oversampling of suspicious cases leading to an overestimation of actual prevalence for *Legionella* infection in SARS-CoV-2 patients may therefore be suspected.

Second, while the parent samples did include around 11,000 SARS-CoV-2 cases, only 46.0% of them were assessed for *Legionella* infection. In fact, some studies substantially assessed the sample as a whole [42,45,48,49,53,62], therefore restraining a still-significant selection bias. However, in most cases, a clear sampling strategy was neither described nor retrospectively deducible through the analysis of the original report. Moreover, even in studies with a larger sampling for *Legionella* infections, it remains unclear how representative the parent population would be of SARS-CoV-2 infections in the general population.

Third, the comparison of prevalence rates across various studies is further complicated by the methodologies of laboratory assessment. Even though most of available studies were based on LUAT, urinary testing is affected by several shortcomings, including an unsatisfying sensitivity and the lack of reliability with pathogens different from *L. pneumophila* serotype 1 [16,92]. On the contrary, in the present estimates, around 25% of index cases were assessed through PCR for a coexisting *Legionella* infection, leading to the diagnosis of one-third of all cases. In other words, testing cases of COVID-19 with LUAT rather than with the more innovative PCR was associated with a reduced likelihood (IRR 0.688, 95% CI 0.622 to 0.760) of new diagnoses, suggesting that a substantial share of actual cases could have been improperly dismissed. In this regard, the referral to PCR in daily practice remains otherwise limited. According to national reports for 2020, PCR was the diagnostic procedure in only 16% of notified cases in France [11], 15% in Germany [10], and 1.4% in Italy [12], i.e., a far lesser share than that identified in our review. As a consequence, we cannot rule out that the very high prevalence of *Legionella* among SARS-CoV-2 patients may be influenced by the “true” occurrence of this pathogen in the general population. However, as the CFR in studies where the positivity for *Legionella* was assessed by means of PCR was substantially higher than in those based on LUAT (i.e., 14.7% vs. 11.6%; RR 1.510, 95% CI 1.277 to 1.787, *p* < 0.001) (See Appendix A Figure A1 and Figure A2), the underlying oversampling of more severe cases, with a baseline increased risk of *Legionella* infection, cannot be ruled out.

## 5. Conclusions

In summary, the collected studies suggest that co-infections by *Legionella* spp. in SARS-CoV-2 patients may be quite less frequent than suggested by early reports, particularly when compared to other pathogens. However, because of the characteristics of the studies, we were able to retrieve, we cannot rule out that the occurrence of infections may be extensively overestimated. In addition, the potential case fatality ratio may have been inflated by the study design, leading to the oversampling of more complicated, and therefore more severe cases of co-infections. However, as *Legionella* remains a substantial public health threat, and NPI implemented to avert SARS-CoV-2 infections are substantially useless against a pathogen that has no inter-human spreading, our data stress the urgent need for higher-quality and specifically designed studies aimed to properly characterize the actual burden of disease. In the meantime, physicians managing SARS-CoV-2-infected patients from high-risk settings for *Legionella* infections (i.e., travel and hospitals) should maintain a high suspicion index for potential co-infections.

## Figures and Tables

**Table 1:** Summary of the case-control studies, cross-sectional studies, and cohort studies included in the meta-analysis. Notes: EU = European union, LD = Legionnaires’ disease; ICU = intensive care unit; LUAT = *Legionella* urinary antigen test; PCR = polymerase chain reaction; n.a. not available.

Study	Year	Country	Timeframe	Setting	Total Sample (No.)	ICU (No., %)	Age (Years, Median)	Males (%)	Sampled for LD (No., %)	Methods	Design	Positive Cases (No., %)
Adler et al. [40]	2020	UK	March–April 2020	ICU + normal ward	195	n.a.	n.a.	n.a.	31, 15.9%	LUAT	Retrospective	1, 0.5%
Baskaran et al. [41]	2021	UK	March–May 2020	ICU	254	254, 100%	59	64.5%	254, 100%	LUAT	Retrospective	3, 0.5%
Chaudhry et al. [42]	2022	India	June 2020–January 2021	ICU + normal ward	194	n.a.	50	71.7%	194, 100%	LUAT	Retrospective	0, -
Chen et al. [44]	2021	China	January–March 2020	ICU + normal ward	408	n.a.	61	55.9%	45, 11,0%	PCR	Retrospective	0, -
Coenen et al. [46]	2021	Netherlands	February–March 2020	ICU + normal ward	281	46, 16.4%	69	62.1%	35, 12.5%	LUAT	Retrospective	0, -
Cohen et al. [72]	2021	Israel	June 2020–March 2021	ICU + normal ward	887	101, 11.4%	69	86.4%	115, 13.0%	PCR	Retrospective	1, 0.9%
Contou et al. [23]	2020	France	March–April 2020	ICU	92	100%	61	68.8%	88, 95.7%	PCR	Retrospective	0, -
He et al. [58]	2020	China	February 2020	ICU + normal ward	194	n.a.	70	64.0%	194, 100%	PCR	Retrospective	0, -
Hughes et al. [26]	2020	UK	February–March 2020	ICU + normal ward	836	n.a.	64	64.3%	246, 29.4%	LUAT	Retrospective	0, -
Husain et al. [60]	2021	France	February–April 2020	Only non ICU	784	n.a.	64	71.7%	491, 62.4%	LUAT	Retrospective	0, -
Ishiguro et al. [57]	2021	Japan	February 2020–January 2021	ICU + normal ward	298	n.a.	61	57.5%	298, 100%	LUAT	Retrospective	2, 0.7%
Karami et al. [61]	2021	Netherlands	March–May 2020	ICU + normal ward	925	n.a.	65	75.6%	199, 21.5%	LUAT	Retrospective	0, -
Kolenda et al. [51]	2020	France	March–April 2020	ICU	99	100%	66	69.0%	99, 100%	PCR	Prospective	1, 1.0%
Paparoupa et al. [53]	2021	Germany	March–November 2020	ICU	102	100%	65	71.0%	102, 100%	PCR	Retrospective	0, -
Rothe et al. [48]	2021	Germany	February–April 2020	ICU + normal ward	140	106, 40.0%	69	64.7%	140, 100%	LUAT	Retrospective	0, -
Rothe et al. [49]	2021	Germany	March 2020–March 2021	ICU + normal ward	200	47, 23.5%	68	61.2%	200, 100%	PCR	Retrospective	3, 1.5%
Rouzé et al. [24]	2021	EU (various countries)	February–March 2020	ICU	568	100%	59	73.0%	55, 9.7%	LUAT	Prospective	0, -
Scott et al. [52]	2022	USA	February–May 2020	ICU + normal ward	1389	327, 23.5%	61	79.0%	1050, 75.6%	LUAT	Retrospective	5, 0.4%
Søgaard et al. [50]	2021	Switzerland	February–May 2020	ICU + normal ward	220	41, 18.6%	n.a.	n.a.	162, 73.6%	LUAT	Retrospective	0, -
Soto et al. [54]	2021	Peru	Septmber–December 2020	ICU + normal ward	93	29, 31.1%	62	71.0%	69, 74.2%	PCR	Retrospective	0, -
Sreenath et al. [43]	2021	India	June 2020–January 2021	ICU + normal ward	191	139, 72.8%	48	48.0%	191, 100%	PCR	Retrospective	1, 0.5%
Townsend et al. [55]	2020	Ireland	March–April 2020	ICU + normal ward	117	40, 34.2%	45	51.0%	84, 71.8%	LUAT	Retrospective	0, -
Verhasselt et al. [62]	2021	Germany	March–July 2020	ICU + normal ward	93	40, 43.0%	63	66.7%	93, 100%	LUAT	Retrospective	1, 1.1%
Wang et al. [56]	2021	UK	March–April 2020	ICU + normal ward	1396	226, 16.2%	59	63.5%	308, 22.1%	LUAT	Retrospective	0, -
Yang et al. [63]	2021	China	January–April 2020	ICU	20	100%	50	42.6%	20, 100%	PCR	Retrospective	0, -
Zamora-Cintas et al. [59]	2021	Spain	March–May 2020	ICU + normal ward	703	15, 7.7%	61	45.0%	15, 2.1%	LUAT	Retrospective	0, -
Zhu et al. [45]	2020	China	February–March 2020	ICU + normal ward	257	17, 6.6%	51	53.7%	257, 100%	PCR	Retrospective	0, -

**Table 2:** Comparison of the prevalence rate for the carriage of *Legionella pneumophila* between the settings and design of the study. Notes: RR = Rate Ratio; 95% CI = 95% Confidence Intervals; ICU = intensive care unit; LUAT = *Legionella* urinary antigen test; PCR = polymerase chain reaction.

	Sampled Population (No. 10,936, %)	No. of Studies (No. 27, %)	No. of Samples (No. 5035, %)	Positive Cases (No. 18, %)	Prevalence of *L. pneumophila* RR (95% CI)
Timeframe					
first semester, 2020	9073, 83.0%	21, 77.8%	3968, 78.8%	11, 61.1%	1.000 (REFERENCE)
Subsequent months	1863, 17.0%	6, 22.2%	1067, 21.2%	7, 38.9%	6.508 (1.909; 22.190)
Area					
European Countries	7005, 64.1%	17, 63.0%	2602, 51.7%	9, 50.0%	1.000 (REFERENCE)
United States	1389, 12.7%	1, 3.7%	1050, 20.0%	5, 27.8%	1.377 (0.462; 4.098)
China	879, 8.0%	4, 14.8%	516, 10.2%	0, -	0.280 (0.016; 4.825)
India	385, 3.5%	2, 7.4%	385, 7.6%	1, 5.6%	0.750 (0.095; 5.911)
Japan	298, 2.7%	1, 3.7%	298, 5.9%	2, 11.1%	1.940 (0.421; 8.938)
Israel	887, 8.1%	1, 3.7%	115, 2.3%	1, 5.6%	2.514 (0.321; 19.676)
Peru	93, 0.9%	1, 3.7%	69, 1.4%	0, -	2.095 (0.122; 35.776)
Clinical Setting					
ICU only	1135, 10.4%	6, 22.2%	618, 12.3%	4, 22.2%	1.000 (REFERENCE)
ICU + non intensive settings	9017, 82.5%	20, 74.1%	3926, 78.0%	14, 77.8%	0.551 (0.182; 1.668)
Non intensive settings only	784, 7.2%	1, 3.7%	491, 9.8%	0, -	0.157 (0.008; 2.969)
Study design					
Retrospective	10,269, 93.9%	25, 92.6%	4881, 96.9%	17, 94.4%	1.000 (REFERENCE)
Prospective	667, 6.1%	2, 7.3%	154, 3.1%	1, 5.6%	1.864 (0.250; 13.920)
Diagnostic procedure					
LUAT	8396, 76.7%	16, 59.3%	3655, 72.6%	12, 66.7%	1.000 (REFERENCE)
PCR	2543, 23.3%	11, 40.7%	1380, 27.4%	6, 33.3%	1.324 (0.497; 3.521)

**Table 3:** Incidence rate ratios (IRR) for Legionnaires’ Disease (LD) cases among SARS-CoV-2 diagnoses by settings of the diagnoses. IRR were calculated by means of a Poisson logistic regression, the incident cases of LD assuming as outcome variables. Note: 95% CI = 95% Confidence Intervals; ICU = intensive care unit; PCR = polymerase chain reaction.

	Total Incident Cases	
	IRR	95% CI
Studies performed during the “First Wave”	0.796	0.735; 0.863
Studies performed outside of Europe	0.609	0.564; 0.658
Diagnosis through Urinary Antigens	0.688	0.622; 0.760
Studies on ICU only	9.009	6.923; 11.725
Median Age (+1 year)	1.055	1.047; 1.062
Male gender (+1%)	1.119	1.112; 1.125
Sampling rate over incident cases (+1%)	1.042	1.039; 1.044

**Table 4:** Summary of the characteristics of individual cases of SARS-CoV-2 and Legionnaires’ disease co-infections.

Variable	No. 19, %	Average ± SD
Male gender	16, 84.2%	
Age (years)		61.9 ± 16.1
Age ≥ 65 years	11, 57.9%	
Country of origin		
France	7, 36.8%	
Japan	2, 10.5%	
UK	2, 10.5%	
Chile	1, 5.3%	
Italy	1, 5.3%	
Germany	1, 5.3%	
Portugal	1, 5.3%	
Saudi Arabia	1, 5.3%	
Spain	1, 5.3%	
USA	1, 5.3%	
Settings of the infection, SARS-CoV-2 *		
Community	13, 68.4%	
Travel	3, 15.8%	
Hospital	2, 10.5%	
Undefined	2, 10.5%	
Settings of the infection, LD *		
Community	13, 68.4%	
Travel	3, 15.8%	
Hospital	2, 10.5%	
Undefined	1, 5.3%	
Other	2, 10.5%	
Admission to the ICU	16, 84.2%	
Length of stay in ICU (days) **		17.7 ± 8.0
Intubation **	7, 36.8%	
Length of intubation (days) **		12.9 ± 7.6
Risk factors		
Obesity	3, 15.8%	
Hypertension	4, 21.1%	
Steroid Therapy	5, 26.3%	
Smoking History	5, 26.3%	
Cardiovascular Disease	6, 31.6%	
Chronic Respiratory Disease	1, 5.3%	
Chronic Kidney Disease	2, 10.5%	
Diabetes	4, 21.1%	
Cancer	3, 15.7%	
Asthma	2, 10.5%	
Rheumatoid Arthritis	1, 5.3%	
Number of Risk Factors > 1	15, 78.9%	
Outcome		
Discharge	9, 47.4%	
Ongoing	5, 26.3%	
Death	5, 26.3%	

* In cases of multiple exposure, all iterations were reported. Therefore, the sum may exceed 100%. ** For 7 cases, no information about the length in ICU nor the eventual intubation have been made available.

**Table 5:** Association of the characteristics of individual cases of SARS-CoV-2 and *Legionella* co-infections with the outcome of being admitted to an intensive care unit (ICU) and death. P-value from Fisher’s exact test.

Variable	ICU (No. 16, %)	*p* Value	Death (No. 5, %)	*p* Value
Male gender	13, 81.3%	1.000	3, 60.0%	0.155
Age ≥ 65 years	10, 62.5%	0.546	5, 100%	0.045
European Area	11, 68.8%	1.000	4, 80.0%	1.000
Community Settings of SARS-CoV-2 infection	10, 62.5%	0.517	3, 60.0%	1.000
Community Settings of LD infection	10, 62.5%	0.517	3, 60.0%	1.000
Risk factors				
Obesity	3, 18.8%	1.000	0, -	0.530
Hypertension	4, 25.0%	1.000	2, 40.0%	0.272
Steroid Therapy	5, 31.3%	0.530	2, 40.0%	0.570
Smoking History	5, 31.3%	0.530	1, 20.0%	1.000
Cardiovascular Disease	6, 37.5%	0.517	2, 40.0%	1.000
Chronic Respiratory Disease	1, 6.3%	1.000	0, -	1.000
Chronic Kidney Disease	2, 12.5%	1.000	2, 40.0%	0.058
Diabetes	3, 18.8%	0.530	1, 20.0%	1.000
Cancer	3, 18.8%	1.000	1, 20.0%	1.000
Asthma	2, 12.5%	1.000	1, 20.0%	0.468
Rheumatoid Arthritis	1, 6.3%	1.000	0, -	1.000
Any Risk Factor	14, 87.5%	0.097	5, 100%	0.530

## Data Availability

The data presented in this study are available on request from the corresponding author.

## Appendix A

**Table A1:** Summary of case reports on SARS-CoV-2 and Legionnaires’ disease (LD) co-infection. Note: ICU = intensive care unit; CVD = cardiovascular disease; CKD, chronic kidney disease; na = not available.

Reference	Country	Timeframe	Gender	Age (Years)	Settings (SARS-CoV-2)	Setting (LD)	Underlying Disease (Any)	ICU (Days)	Intubation (Days)	Outcome
Alhuofie, S. [64]	Saudi Arabia	June 2020	Male	76	Community	Community	Diabetes	No	-	Discharge
Allam et al. [35]	France	March 2020	Male	72	Hospital	Hospital	CVD, Cancer	Yes (na)	na	Ongoing
			Male	71	Hospital Travel	Hospital Travel	Smoking, CVD, CRD, Diabetes,	Yes (na)	na	Ongoing
			Male	71	Community	Community	Steroid therapy, Smoking, CVD, Cancer	Yes (na)	na	Death
			Female	83	Community	Community	Steroid therapy, CVD	Yes (na)	na	Discharge
			Male	73	Community	Plumbing	CVD, Diabetes	Yes (na)	na	Ongoing
			Male	73	Community	Community	CVD, CKD	Yes (na)	na	Death
			Male	37	Community	Community	-	Yes (na)	na	Ongoing
Anderson et al. [65]	USA	April 2020	Male	49	Community	Community	-	Yes (10)	Yes (10)	Discharge
Arashiro et al. [28]	Japan	March 2020	Male	80	Travel	Travel	Diabetes	Yes (23)	Yes (10)	Death
Argemí et al. [66]	Spain	November 2020	Male	35	Community	Community	-	Yes (9)	-	Discharge
Camoes et al. [67]	Portugal	November 2020	Male	53	Undefined	Community	Obesity, Smoking	Yes (20)	Yes (10)	Discharge
Chalker et al. [68]	UK	February 2020	Female	65	Undefined	Undefined	Asthma, Hypertension, Steroid therapy,	Yes (20)	Yes (20)	Death
		April 2020	Female	80	Community	Community	Hypertension, CKD	Yes (5)	Yes (5)	Death
Choappa et al. [69]	Chile	July 2020	Male	47	Community	Community	Obesity, Hypertension, Smoking, previous COVID-19	Yes (27)	Yes (27)	Discharge
Palazzolo et al. [29]	Italy	June 2020	Male	40	Community	Community	-	No	-	Discharge
Shimizu et al. [70]	Japan	Na.	Male	73	Travel	Travel Hot Tubes	Cancer	Yes (27)	Yes (8)	Discharge
Subedi and Haas [71]	USA	Na.	Male	58	Community	Community	Rheumatoid Arthritis, Obesity, Hypertension, Abuse of Opiate, Steroid therapy, Smoking	Yes (18)	-	Discharge
Verhasselt et al. [62]	Germany	March 2020	Male	41	Community	Community	Asthma, Steroid therapy	Yes (na)	Yes (na)	Ongoing
